# Promising influences of hesperidin and hesperetin against diabetes and its complications: a systematic review of molecular, cellular, and metabolic effects

**DOI:** 10.17179/excli2023-6577

**Published:** 2023-12-04

**Authors:** Amirhossein Mirzaei, Ali Mirzaei, Shakiba Najjar Khalilabad, Vahid Reza Askari, Vafa Baradaran Rahimi

**Affiliations:** 1 Student Research Committee, Faculty of Medicine, Mashhad University of Medical Sciences, Mashhad, Iran; 2 Applied Biomedical Research Center, Mashhad University of Medical Sciences, Mashhad, Iran; 3 Pharmacological Research Center of Medicinal Plants, Mashhad University of Medical Sciences, Mashhad, Iran; 4 International UNESCO Center for Health-Related Basic Sciences and Human Nutrition, Mashhad University of Medical Sciences, Mashhad, Iran; 5 Department of Cardiovascular Diseases, Faculty of Medicine, Mashhad University of Medical Sciences, Mashhad, Iran

**Keywords:** hesperidin, hesperetin, diabetes, diabetes complications, inflammation, oxidative stress

## Abstract

Hesperidin and hesperetin, two flavonoids with potential therapeutic value, have been extensively studied in the context of diabetes management. The main objective of this research is to ascertain their potential as therapeutic options for managing diabetes and its complications. The present study utilized a systematic review methodology and comprehensively explored relevant literature from databases, including PubMed, Scopus, and Web of Science, from inception until July 2023. The review summarized the outcomes related to the molecular, cellular, and metabolic effects of hesperidin and hesperetin in diabetes and its complications. Hesperetin exhibits a potential treatment for preventing diabetes and its associated complications through modulation of inflammatory cytokine release and expression via the pathway of signaling through Toll-like receptor/Myeloid differentiation factor 88/Nuclear factor-kappa B. Hesperidin shows promise as a biomolecule for treating diabetic neuropathy, primarily through activation of nuclear factor erythroid 2-related factor 2 (Nrf-2), as an antioxidant-response element signaling, leading to neuroprotective effects. Both compounds demonstrated the ability to normalize blood glucose levels and reduce serum and liver lipid levels, making them potential candidates for managing hypoglycemia and hypolipidemia in diabetes. Hesperidin also showed potential benefits against diabetic nephropathy by suppressing transforming growth factor-β1-integrin-linked kinase-Akt signaling and enhancing renal function. Furthermore, hesperidin's antioxidant, anti-inflammatory, and anti-depressant effects in diabetic conditions expanded its potential therapeutic applications. This systematic review provides substantial evidence supporting the consideration of hesperidin and hesperetin for diabetes and its complications. It offers exciting possibilities for developing novel, cost-effective treatment options to enhance diabetes management and patient outcomes.

## Abbreviations

5-HT5-hydroxytryptamine

8-OhdG 8-hydroxy-20-deoxyguanosine

AchE Acetyl Cholinesterase

ACVRL1 Activin A receptor-like type 1

ADAM9 A disintegrin and a metalloproteinase 9

AGEs Advanced glycation end products

AIP Atherogenic index of plasma

ALP Alkaline phosphatase

ALT Alanine aminotransferase

AMPK AMP (adenosine monophosphate) Activated protein kinase

Ang-1 Angiopoietin-1

AR Aldose reductase

AREAntioxidant-response element

AST Aspartate aminotransferase

b.w. Body Weight

BDNF Brain-derived neurotrophic factor

BUN Blood urea nitrogen

CAT Catalase

CD Cyclodextrin

CES Cholesterol ester synthetase 

CHOP CCAAT-enhancer-binding protein (C/EBP) homologous protein

ChREBP carbohydrate response element-binding protein

CK Creatine kinase

CK-MB Creatine kinase myoglobin binding

CNS Central nervous system

CoIV Type IV collagen

COX-2 Cyclooxygenase-2

CREB cAMP response element-binding protein

CRP C-reactive protein

DA Dopamine

DAP Diastolic arterial pressure

DM Diabetes mellitus

DN Diabetic nephropathy

DNP Diabetic neuropathy

ECs Endothelial cells

eIF2-α Eukaryotic initiation factor 2 subunit alpha

EPM Elevated plus maze 

ER Endoplasmic reticulum

ERK Extracellular signal−regulated kinase

FBG Fasting blood glucose

FBS Fasting blood sugar

FGF-23 Fibroblast growth factor-23

FINSFasting insulin

FN Fibronectin

FST Forced swimming test

GDM Gestational diabetes mellitus

GK Glucokinase

Glo-1 Glyoxalase 1

GOT Glutamate oxaloacetate transferase

GPx Glutathione peroxidase

GR Glutathione Reductase

GRP78 Glucose-regulated protein 78

GSH Glutathione

GSH-Px Glutathione peroxidase

GSI Gonadosomatic index

GSK-3β Glycogen synthase kinase-3β

GST Glutathione-S-transferase

H&E Hematoxylin and eosin

HAEC Human aortic endothelial cell

HbA1c Glycated hemoglobin

HBT Hole board test

HDL High-density lipid-protein

HDP Hydroxyproline

HG High glucose

HMG-CoAreductase Hydroxymethylglutaryl-CoA reductase

HO-1 Heme oxygenase-1

HOMA-IR Homeostasis model of insulin resistance

I/R Ischemia and reperfusion

ICAM-1 Intercellular adhesion molecule-1

ICR mice Institute of Cancer Research mice

IL-1β Interleukin-1 beta

IL-6 Interleukin-6

ILK Integrin-linked kinase

iNOS inducible nitric oxide synthase

IP Intraperitoneal injection

IR Insulin receptor

IRS-1 Insulin receptor substrate 1

ISO Isoproterenol

ITGAV Integrin α-V

JNK Jun N-terminal kinases

Kv Voltage-dependent K+

LCAT Lecithin Cholesterol acyl transferase

LDH Lactate dehydrogenase

LDL Low-density lipid-protein

LPL Lipoprotein lipase

LPO Lipid peroxidation 

LPS Lipopolysaccharide

LVEDP Left ventricular end-diastolic pressure

LVEF Left ventricular ejection fraction

LVFS Left ventricular fractional shortening

MafA v-maf musculoaponeurotic fibrosarcoma oncogene family protein A

MAO Monoamine oxidase

MAP Mean arterial pressure 

MBT Marble burying test

MCEW Mean cauda epididymis weights

MCP-1 Monocyte chemoattractant protein-1

MDA Malondialdehyde

MI Myocardial infarction

MPO Myeloperoxidase

MTW Mean testis weights

MyD88 Myeloid differentiation factor 88

N2a Neuro 2A

NE Norepinephrine

NF-κB Nuclear factor kappa B

NIC Nicotinamide

NO Nitric oxide

Nox2 NADPH oxidase 2

NP-SH Nonprotein sulfhydryls

Nrf2 Nuclear factor erythroid 2-related factor 2

OFT Open field test

OGTT Oral glucose tolerance test

PARP Poly (ADP-ribose) polymerase

PAS Periodic acid-Schiff

PC Protein carbonyl

PCCB Propionyl CoA carboxylase β

PCNA Proliferating cell nuclear antigen

PDK1 Phosphoinositide-dependent kinase 1

PDX-1 Pancreatic-duodenal homeobox-1

PERK Protein kinase R-like endoplasmic reticulum kinase

PGE_2_ Prostaglandin E_2_

PKA Protein kinase A

PPAR Peroxisome proliferator-activator receptor

QFA Quzhou *Fructus aurantii*

QUICKI Quantitative insulin sensitivity check index

RAGE Receptor for advanced glycation end products

RCA Rat coronary artery

RCASMC Rat coronary arterial smooth muscle cell

RNA pol II RNA polymerase II

ROS Reactive oxygen species

SAP Systolic arterial pressure 

SIRT Sirtuin

SMAD Suppressor of Mothers against Decapentaplegic

SOCS-3 Suppressor of cytokine signaling-3

SOD Superoxide dismutase

STZ Streptozotocin

T2DM Type 2 diabetes mellitus

TAC Total antioxidant capacity

TBARS Thiobarbituric acid reactive substances

TC Total cholesterol

TG Triglycerides

TGFBR2 Transforming growth factor-β receptor type 2

TGF-β1 Transforming growth factor-β1

TLR Toll-like receptor

TNF-α Tumor necrosis factor-alpha

TRPM2 Transient receptor potential melastatin 2

TUNEL Terminal deoxynucleotidyl transferase dUTP nick end labeling

TXNIP Thioredoxin-interacting protein

VCAM1 Vascular cell adhesion molecule 1

VEGF Vascular endothelial growth factor

VEGFR Vascular endothelial growth factor receptors

VLDL Very low-density lipid-protein

XO Xanthine oxidase

α-KL Alpha-klotho

α-SMA Alpha-smooth muscle actin 

γ-GCS Gamma-glutamylcysteine synthetase

## Introduction

Diabetes, a chronic condition resulting from insufficient insulin production or utilization, presents a major global health challenge, with potential complications such as cardiovascular diseases (Gholoobi et al., 2021[24]), nerve damage, kidney damage, lower-limb amputation, and eye diseases (Rakhshandeh et al., 2022[41]). Type 1 diabetes, prevalent in childhood, necessitates lifelong insulin therapy for survival (Vanderniet et al., 2022[51]). On the other hand, type 2 diabetes, responsible for over 90 % of global cases, offers the potential for prevention and delay (Majety et al., 2023[37]). Prediabetes identifies individuals at higher risk for type 2 diabetes and related complications (Roohbakhsh et al., 2020[45]). Additionally, gestational diabetes poses risks to both mothers and babies during pregnancy and birth (Sweeting et al., 2022[50]).

Approximately 537 million adults aged 20-79 live with diabetes, accounting for about 10.5 % of this age group worldwide. Disturbingly, projections indicate a steady rise in prevalence, with estimates forecasting 643 million affected individuals (11.3 %) by 2030 and a further escalation to 783 million (12.2 %) by 2045 (Russo et al., 2023[46]). Undiagnosed diabetes presents a significant challenge, affecting around 240 million people globally, with higher prevalence in countries with low- and middle-income (Yoshida et al., 2023[59]). Proper management of diabetes is crucial to prevent complications and improve overall health (Akhlaghipour et al., 2023[6]). 

**Figure 1:**
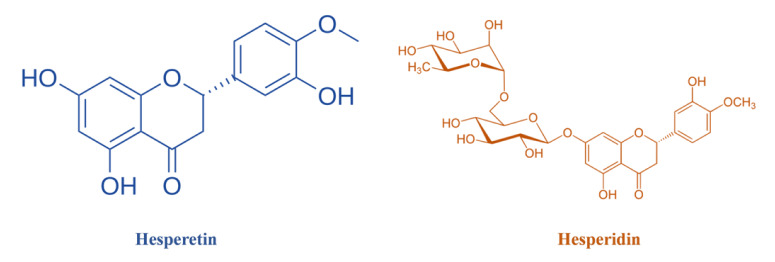
Molecular configuration of hesperidin and hesperetin

Hesperidin and hesperetin (Figure 1(Fig. 1)), two potent bioactive compounds inherent to citrus fruits, display impressive antioxidant capabilities and wield a diverse range of biological effects, holding substantial promise for both preventing and treating various diseases (Franke et al., 2018[21]; Iskender et al., 2017[26]; Khorasanian et al., 2023[31]). Hesperidin, identified as hesperetin 7-rhammnoglucoside, represents a glycoside variation of hesperetin and is a notable flavanone present in significant quantities within fruits such as tangerines, lemons, oranges, limes, and grapefruits (Askari et al., 2020[11]; Rahmani et al., 2023[40]). The hesperidin content ranges from 20-60 mg in oranges, 8-46 mg in tangerines, 4-41 mg in lemons, and 2-17 mg in grapefruits per 100 mL of juice. The peel's outer and soft middle layers contain more hesperidin than hand-squeezed juice, making commercial juices with peel components a rich source (Ghadiri et al., 2021[23]). Additionally, hesperidin is present not only in citrus fruits but also in mint plants, honeybush, and flavored tea (Garg et al., 2001[22]; Pyrzynska, 2022[39]). These encompass the modulation of oxidative stress, reduction of inflammation, facilitation of nitric oxide synthesis, control of hypertension, combatting infections, and regulation of apoptosis (Buzdağlı et al., 2022[13]). Its multifaceted impact positions hesperidin as a compelling candidate for addressing conditions such as cardiovascular disorders, metabolic syndrome, non-alcoholic fatty liver disease (NAFLD), and insulin resistance (Khorasanian et al., 2023[31]; Shams-Rad et al., 2020[47]).

Meanwhile, hesperetin, another noteworthy flavanone, is notably present in citrus fruits such as grapes, lemons, and oranges, primarily appearing as hesperidin in the peel of *Citrus aurantium* L. (Evans et al., 2022[20]). Upon ingestion, hesperidin undergoes conversion into its bioactive form, hesperetin, a pivotal transformation that underscores its potential applications. Noteworthy advantages of hesperetin include ease of extraction, consistent and stable biological activity, and a spectrum of benefits ranging from antioxidant potency and cardiovascular modulation to safeguarding the nervous system, mitigating allergic responses, inhibiting microbial activity, and even showing potential anti-cancer properties (Kumar et al., 2017[32]).

This systematic review analyzes the literature to provide a comprehensive overview of the therapeutic potential of hesperidin and hesperetin in diabetes management. By exploring their molecular, cellular, and metabolic effects, the review aims to enhance understanding of their pharmacological properties and implications for diabetes treatment. The synthesis may guide the development of targeted interventions to improve diabetes management and patient outcomes. 

## Methods

The research question for this systematic review is to assess the molecular, cellular, and metabolic effects of hesperidin and hesperetin use in diabetes. The systematic review protocol was developed to outline the objectives and methods of the review, including the selection criteria for studies. Databases including Scopus, PubMed, and Web of Science from Incept to July 2023 were utilized to conduct a comprehensive literature search. The search terms and keywords used to identify relevant studies included hesperidin, hesperetin, Diabetes, Diabetic, Complications of Diabetes Mellitus, Diabetes Complication, Diabetes Complications, Diabetes Mellitus Complication, Diabetes Mellitus Complications, Diabetes Mellitus Experimental, Diabetes Mellitus Lipoatrophic, Diabetes Mellitus Type 1, Diabetes Mellitus Type 2, Diabetes-Related Complications, Diabetes Gestational, Diabetes-Related Complication, Diabetes-Related Complications, Diabetic Angiopathies, Diabetic Cardiomyopathies, Diabetic Coma, Diabetic Complication, Diabetic Complications, Diabetic Foot, Diabetic Ketoacidosis, Diabetic Nephropathies, Diabetic Neuropathies, Diabetic Retinopathy, Diet Diabetic, Donohue Syndrome, Fetal Macrosomia, Gastroparesis, Glucose Intolerance, Glycation End Products Advanced, Hyperglycemic Hyperosmolar Nonketotic Coma, Latent Autoimmune Diabetes in Adults, Prediabetic State, Scleredema Adultorum, and Wolfram Syndrome.

The method of choosing studies relied on predetermined criteria for inclusion and exclusion. The research was incorporated if it explored the impacts of hesperidin and hesperetin within the diabetes context, involving molecular, cellular, or metabolic outcomes. The inclusion criteria encompassed studies published in English, original research articles, and those available in full text. Exclusion criteria involved studies that were review articles, book chapters, those written in languages other than English, and those with no access to full-text articles. 

**Figure 2:**
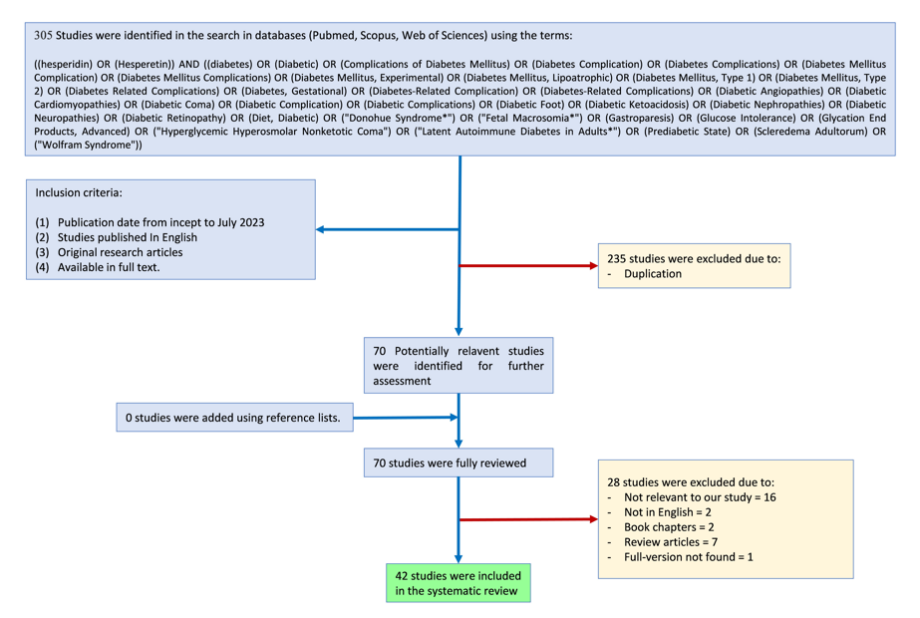
The flowchart of the study

The initial search yielded a total of 305 articles. After removing duplicates (235), non-English articles (2), book chapters (2), and review articles (7), there were 59 articles remaining. After screening titles and abstracts for relevance, 16 articles were deemed irrelevant and excluded. Additionally, 1 article could not be accessed in full text. Finally, 42 articles met our inclusion criteria and were included in the final review (Figure 2(Fig. 2)). Data was extracted from each included study using a standardized data extraction form. The extracted information included study characteristics (e.g., authors, publication year, research structure), number of participants, traits of the participants, treatments (amount and length of hesperidin and hesperetin application), outcomes assessed (molecular, cellular, and metabolic effects), and any relevant findings or results. Due to the heterogeneity of study designs and outcomes, a narrative synthesis approach was used to summarize the findings. The results were categorized based on the molecular, cellular, and metabolic effects of hesperidin and hesperetin in diabetes.

## Results

**Figure 3:**
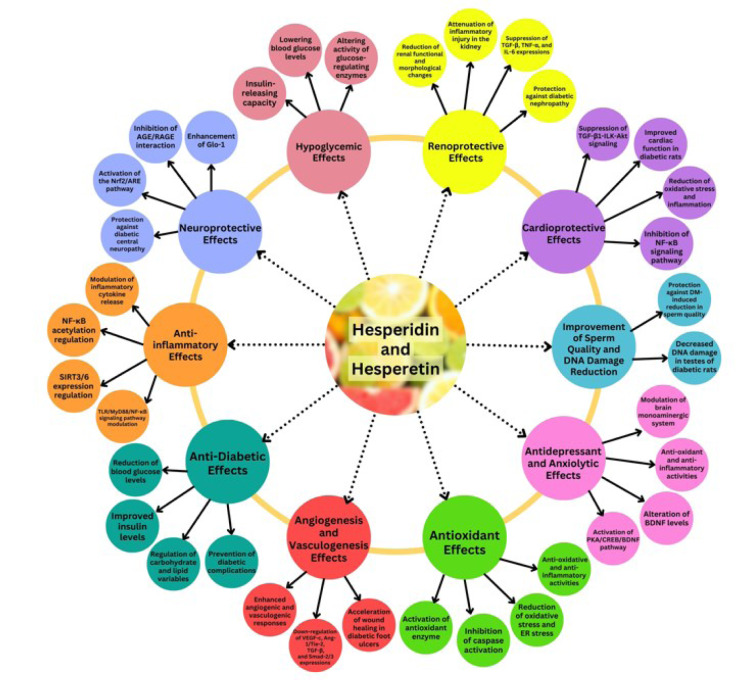
The anti-diabetic effects of hesperidin and hesperetin

The main anti-diabetic mechanisms and effects of hesperidin and hesperetin are illustrated in Figure 3(Fig. 3).

### In vitro models of diabetes mellitus 

Lee and coworkers investigated the anti-inflammatory effects of hesperetin, specifically in the inflammation associated with diabetes. THP1 macrophages were used to simulate diabetic conditions at the cellular level. They were exposed to lipopolysaccharide (LPS) under hyperglycemic conditions, and the researchers examined how hesperetin influenced various aspects of inflammation. The findings indicated that hesperetin, across concentrations spanning 0 to 100 μM over 48 hours, had no detrimental effects on cell viability, indicating its safety. Under hyperglycemic conditions with LPS stimulation, the levels of pro-inflammatory cytokines tumor necrosis factor-α (TNF-α) and interleukin-6 (IL-6) increased. However, hesperetin treatment effectively reduced these elevations, indicating its potential to alleviate inflammation in diabetes (Lee et al., 2021[33]).

Furthermore, hesperetin inhibited key signaling pathways involved in inflammation, specifically Toll-like receptor (TLR) pathways, including TLR2/4 and myeloid differentiation factor 88 (MyD88). Additionally, hesperetin suppressed the expression and nuclear translocation of nuclear factor-κB (NF-κB), further attenuating the inflammatory response. Moreover, hesperetin upregulated the expression of sirtuin (SIRT)3 and SIRT6 proteins, known for their roles in cellular stress response and anti-inflammatory activities. Concentrations ranging from 10 to 50 μM ensured significant anti-inflammatory effects without compromising cell viability. In conclusion, these results imply that hesperetin shows potential as a viable treatment option for handling inflammation linked to diabetes, presenting a novel avenue for addressing diabetic complications through its anti-inflammatory properties (Lee et al., 2021[33]).

In another study, the researchers aimed to explore the safeguarding impacts of hesperetin and naringenin on pancreatic β cells when exposed to elevated glucose levels. They conducted experiments using a cell line derived from pancreatic β cells known as INS-1 as an *in vitro* model. The cells were investigated to assess their impact and received therapeutic intervention using 100 μM hesperetin for 24 hours. The outcomes indicated that hesperetin and naringenin successfully protected pancreatic β cells against apoptosis triggered by elevated glucose levels. This protective effect was not solely reliant on their direct antioxidant capacity. Instead, the compounds were found to inhibit histone acetylation, specifically H3K18 and H3K27, in a dose-dependent manner. Notably, high glucose exposure enhanced histone acetylation in pancreatic β cells (Wang et al., 2021[53]).

Moreover, naringenin and hesperetin were found to suppress the expression of Thioredoxin-interacting protein (TXNIP), an essential regulator of high glucose-induced pancreatic β cell apoptosis, by preventing the attachment of carbohydrate response element-binding protein (ChREBP) to the Txnip promoter region. The study also revealed that naringenin and hesperetin inhibited histone acetylation through AMP (adenosine monophosphate) activated protein kinase (AMPK)-mediated p300 inactivation. By enhancing AMPK activity, these compounds reduced p300's acetylating activity, decreasing histone acetylation and TXNIP expression. In conclusion, the results highlighted the potential of naringenin and hesperetin as protective agents for pancreatic β cells under high glucose conditions. Using 100 μM hesperetin in the *in vitro* experiments provided valuable insights into its protective effects on pancreatic β cells when exposed to high glucose levels for 24 hours (Wang et al., 2021[53]).

Chae and Shin investigated the potential of different concentrations of hesperidin to ameliorate insulin resistance provoked by inflammation within the adipose tissue. The research utilized cells of the RAW 264.7 lineage and mature adipocytes of the 3T3-L1 cell line, which received prior treatment with various doses of hesperidin (5, 50, and 250 μM for RAW 264.7 cells and 50 and 100 μM for differentiated 3T3-L1 adipocytes) before exposure to inflammatory stimuli (TNF-α or LPS). The findings demonstrated that hesperidin effectively suppressed the synthesis of inflammatory markers such as IL-6, TNF-α, and nitric oxide (NO) in a dose-dependent manner in RAW 264.7 cells. Additionally, hesperidin suppressed TNF-α-induced production of IL-6 and prostaglandin E_2_ (PGE_2_) in 3T3-L1 adipocytes while upregulating the mRNA levels of adiponectin and peroxisome proliferator-activator receptor (PPAR)-γ, crucial components affecting insulin sensitivity. These findings suggest that hesperidin may hold promise in mitigating inflammation-mediated insulin resistance in adipose tissue (Table 1(Tab. 1); References in Table 1: Chae and Shin, 2012[15]; Lee et al., 2021[33]; Wang et al., 2021[53]) (Chae and Shin, 2012[15]).

### Animal models of diabetes mellitus 

The promising anti-diabetic effects of** hesperidin and hesperetin** have been reported in different animal models of diabetes mellitus, including streptozotocin (STZ)-induced diabetes mellitus (Table 2(Tab. 2); References in Table 2: Ahmed et al., 2012[5]; Akiyama et al., 2009[8], 2010[7]; Chae and Kim, 2012[14]; Dokumacioglu et al., 2019[17]; Elshazly et al., 2018[19]; Hanchang et al., 2019[25]; Iskender et al., 2017[26]; Peng et al., 2021[38]; Rekha et al., 2019[42]; Revathy et al., 2018[43]; Roghani et al., 2010[44]; Sundaram et al., 2019[49]; Wang et al., 2019[54], 2021[53]).

#### Protective effects of hesperidin and hesperetin against streptozotocin (STZ)-induced diabetes mellitus 

Ahmed and coworkers investigated the effects of hesperidin and naringin on rats with type 2 diabetes induced by a high-fat diet and STZ. The rats were given a daily oral dose of 50 mg/kg body weight of either hesperidin or naringin for 30 days. The findings revealed that hesperidin and naringin effectively reduced elevated glucose levels, lactate dehydrogenase (LDH), glycated hemoglobin (HbA1C), creatine kinase-myoglobin binding (CK-MB), and aspartate aminotransferase (AST) levels while elevating insulin levels in the blood and boosting glycogen content in the liver and muscles of rats with insulin resistance due to diabetes. Moreover, these compounds positively influenced lipid profiles, serum adiponectin, and resistin levels. The study also highlighted the potential of hesperidin and naringin to act as anti-hyperglycemic and antidyslipidemic agents, with favorable effects on cardiac function in diabetic rats. In conclusion, the oral administration of hesperidin and naringin significantly improved various markers of diabetes and cardiac function in diabetic rats (Ahmed et al., 2012[5]). Similarly, hesperetin treatment (10 mg/kg for six weeks) strikingly mitigated serum glucose levels, serum total cholesterol levels, and low-density lipid-protein (LDL) levels in STZ-induced diabetic rats compared (Roghani et al., 2010[44]).

In another study, STZ-induced diabetic rats were treated with hesperetin at 40 mg/kg body weight for 45 days. Hesperetin supplementation led to a notable decrease in blood glucose levels and a substantial enhancement in plasma insulin and glycogen levels. Furthermore, it restored the functioning of hepatic glucose metabolic enzymes and positively influenced lipid profiles. Hesperetin also exhibited antioxidant properties, increasing the levels of antioxidant enzymes both in the bloodstream and pancreas. Additionally, the therapy alleviated kidney and liver toxicity indicators and preserved the normal histological structure of the kidney, liver, and insulin-producing β-cells. The findings suggest that hesperetin effectively alleviates hyperglycemia and dyslipidemia in diabetic rats through its antioxidant properties (Revathy et al., 2018[43]). Furthermore, hesperidin (a diet containing 10 mg/kg) remarkably alleviated blood glucose levels by affecting glucose-regulating enzymes and normalized lipid and adiponectin concentrations in rats with experimentally induced marginal type 1 diabetes using STZ. However, there were no significant changes in bone tissue, bone parameters, or bone metabolic markers due to hesperidin administration in the STZ-injected diabetic rats (Akiyama et al., 2010[7]).

Iskender et al. examined the impact of hesperidin (100 mg/kg body weight for 15 days) on STZ-induced diabetes in rats. In diabetes, antioxidant enzyme activities, namely superoxide dismutase (SOD), sirtuin (SIRT) 1, and catalase (CAT) decrease, resulting in the induction of oxidative stress and subsequent cellular damage. The diabetic group exhibited stimulated levels of oxidative stress markers, malondialdehyde (MDA), and nuclear factor kappa B (NF-kB) in the kidney and liver. The administration of hesperidin notably propagated antioxidant SIRT1, SOD, and CAT activities in kidney tissues, indicating a potential restoration of the body's antioxidant defense mechanism. In addition, hesperidin also attenuated oxidative stress by decreasing NF-kB and MDA levels in the liver and kidney. In conclusion, the study revealed that hesperidin supplementation in diabetic rats enhanced antioxidant activities and reduced oxidative stress markers in liver and kidney tissues (Iskender et al., 2017[26]).

Hesperidin administration (100 mg/kg for two weeks) significantly increased concentrations of alpha-klotho (α-KL) in renal tissues, serum, and liver, indicating a positive effect in countering the decrease observed in the STZ-induced diabetic group. In contrast, levels of fibroblast growth factor-23 (FGF-23), which were elevated in the kidney, and serum of diabetic rats, significantly decreased with hesperidin treatment. Additionally, hesperidin administration was associated with a notable reduction in serum glucose, blood urea nitrogen (BUN), AST, creatinine, and alanine aminotransferase (ALT) levels in diabetic rats, signifying its potential to ameliorate diabetic complications in the liver and kidneys. The findings suggest that hesperidin exhibits promising potential as an alternative treatment for diabetic complications, possibly mediated by modulating the α-KL/FGF-23 pathway (Dokumacioglu et al., 2019[17]).

Additionally, Hanchang et al. explored the potential protective effects of hesperidin (100 mg/kg over four weeks) on pancreatic β-cells in STZ-induced diabetic rats. The administration of hesperidin strikingly reduced levels of fasting blood glucose and intake of food while increasing the weight of the body, levels of insulin in the serum and pancreas, and the pancreatic-duodenal homeobox-1 (PDX-1) expression. Hesperidin also demonstrated antioxidant effects by enhancing SOD and glutathione peroxidase (GPx) activities and reducing nitrotyrosine and MDA levels. Additionally, it reduced TNF-α concentration. It suppressed endoplasmic reticulum (ER) stress markers (Glucose-regulated protein 78 (GRP78) and CCAAT-enhancer-binding protein (C/EBP) homologous protein (CHOP) proteins) while influencing apoptosis-related proteins favorably. In conclusion, hesperidin holds promise in protecting pancreatic β-cells and enhancing their function in diabetes via its anti-apoptotic, anti-inflammatory, and antioxidative mechanisms while mitigating oxidative and ER stress (Hanchang et al., 2019[25]).

Wang and coworkers investigated the protective effects of hesperidin (1.25, 2.5, and 5 mg/kg/day) against gestational diabetes mellitus (GDM) induced by STZ in rat models. The results revealed significant improvements in various GDM-related parameters with hesperidin treatment. Placental and fetal weight, blood glucose levels, lipid profile, glycogen levels, and serum insulin levels were all positively affected dose-dependent. Additionally, hesperidin showed a partial reduction in fetal developmental defects compared to the GDM group. Hesperidin's protective effects were linked to its modulation of the advanced glycation end products (AGEs)/receptor for the advanced glycation end products (RAGE) signaling pathway, which is critical in GDM-related complications. Moreover, it demonstrated antioxidant properties, elevating SOD, glutathione (GSH), GPx, and CAT levels, suggesting enhanced protection against oxidative stress. Furthermore, gene expression analysis indicated that hesperidin downregulated key genes associated with diabetes and hyperglycemia, such as Nicotinamide adenine dinucleotide phosphate (NADPH) oxidase 2 (NOX2), RAGE, monocyte Chemoattractant Protein-1 (MCP-1), p65, and vascular Cell Adhesion Molecule-1 (VCAM-1), potentially mitigating GDM-related complications. In conclusion, hesperidin showed promise in protecting fetal development in GDM-induced pregnant rats by positively impacting various parameters and the AGEs/RAGE signaling pathway. Its antioxidant activity and gene expression modulation suggest its potential therapeutic value in managing GDM and improving neonatal outcomes (Wang et al., 2019[54]).

Another study involved inducing type 2 diabetes in rats using STZ and nicotinamide, followed by an 8-week treatment with hesperidin (100 mg/kg/day) alone or in combination with omeprazole or GW9662, which acts as an antagonist for peroxisome proliferator-activated receptor-γ (PPAR-γ). Subsequently, the acute gastric injury was induced through cold restraint stress. The results demonstrated that hesperidin treatment significantly improved glycemic control, effectively reducing gastric acidity and ulcer indices and ameliorating histopathological changes in gastric mucosa, comparable to omeprazole's effects. Moreover, hesperidin exhibited potent antioxidant properties by reducing oxidative stress markers and enhancing the antioxidant capacity, along with notable anti-inflammatory effects by decreasing inflammatory markers. Furthermore, the study found that hesperidin increased the expression of PPAR-γ in gastric tissues, suggesting the involvement of PPAR-γ activation in mediating these protective effects. However, when GW9662, a PPAR-γ antagonist, was co-administered with hesperidin, it attenuated the beneficial effects of hesperidin, supporting the notion that the protective impact of hesperidin against gastric ulcers is indeed mediated through PPAR-γ activation. In conclusion, the findings demonstrate the potential therapeutic value of hesperidin in managing stress-induced gastric ulcers in diabetic individuals. Hesperidin's ability to modulate oxidative stress and inflammation through PPAR-γ activation makes it a promising candidate for further exploration as a treatment option for gastric ulcers in the context of diabetes (Elshazly et al., 2018[19]).

Hesperidin (25, 50, and 100 mg/kg b.w.) led to a decline in fasting plasma glucose levels in rats that were made diabetic through the use of STZ, and this decrease varied according to the dosage. Besides its role in decreasing plasma glucose levels, hesperidin also elevated hemoglobin and insulin levels while decreasing glycosylated hemoglobin, indicating improved glycemic control. Moreover, hesperidin positively influenced key enzymes involved in carbohydrate metabolism. In the liver, activities of glucose-6-phosphate dehydrogenase and hexokinase were notably enhanced, leading to an augmentation in glucose utilization. Conversely, the activities of fructose-1, 6-bisphosphatase and glucose-6-phosphatase were decreased, leading to reduced glucose production and enhanced glycogen synthesis. Moreover, the application of hesperidin prevented the loss of weight among rats with diabetes and enhanced the glycogen content within the liver. This improvement was attributed to increased glycogen synthase and glycogen phosphorylase activities, essential enzymes regulating glycogen storage and release. Hesperidin demonstrated remarkable anti-hyperglycemic activity, promoting better glucose control and ameliorating diabetes-induced alterations in glucose-metabolizing enzymes (Sundaram et al., 2019[49]).

Additionally, hesperidin (100 mg/kg over a duration of four weeks) markedly diminished blood glucose, total cholesterol, triglycerides, high-density lipid-protein )HDL(, LDL, and Very low-density lipid-protein )VLDL( levels in STZ-induced diabetes mellitus (DM). It demonstrated anti-hyperglycemic and hypo-lipidemic effects without causing body weight loss. Moreover, hesperidin exhibited antioxidant properties and had no adverse effects on cardiovascular risk markers. These findings suggest that hesperidin could be a safe and effective treatment for managing DM in rats, offering potential benefits for diabetic and dyslipidemic conditions (Rekha et al., 2019[42]).

#### Protective effects of hesperidin and hesperetin against other models of DM

Akiyama and coworkers focused on evaluating the impacts of two types of hesperetin glycosides, specifically cyclodextrin (CD)-clathrated hesperetin and hesperidin, on glucose and lipid metabolism in weanling rats of the Goto-Kakizaki (GK) strain, which exhibit type 2 diabetes. The researchers observed that both CD-hesperetin and hesperidin positively impacted the metabolism of glucose through influencing the enzymes involved in glucose regulation. Additionally, they found that these compounds decreased levels of lipids in the liver and serum, indicating the potential effects of reducing lipid levels. Hesperidin, derived from citrus fruits, is known for its various health benefits, including anti-inflammatory, anti-carcinogenic, antioxidant, and lipid-lowering properties. It has been reported to reduce cholesterol levels in the blood and liver. The study showed promising results, suggesting that hesperidin and CD-hesperetin could be therapeutic agents for managing diabetes and dyslipidemia. A comparison between native hesperidin and CD-hesperetin highlighted the potential advantages of CD-clathrated hesperetin, as it exhibited higher water solubility and increased serum levels of hesperetin. These findings have created new paths for potential clinical applications of hesperidin and CD-hesperetin in managing type 2 diabetes and related lipid disorders (Akiyama et al., 2009[8]). 

### Effects of hesperidin and hesperetin on insulin resistance and secretion

Hesperidin was investigated for its potential to mitigate insulin resistance induced by IL-6 in hepatocytes. The study utilized the Hepa-lclc7 cell line and treated the cells with hesperidin at 50 and 100 μM concentrations before exposing them to IL-6. The experimental results revealed that hesperidin effectively restored the reversed reduced insulin receptor substrate 1 (IRS-1) protein expression caused by IL-6, indicating a positive modulation of insulin receptor signaling in the liver cells. Additionally, hesperidin demonstrated the ability to downregulate the expression of suppressor of cytokine signaling-3 (SOCS-3) and C-reactive protein (CRP) mRNA, both of which play pivotal roles in insulin resistance, and it also exhibited inhibitory effects on IL-6 production induced by LPS. In conclusion, hesperidin remarkably ameliorates hepatic insulin resistance induced by IL-6 in hepatocytes. The restoration of IRS-1 expression and the downregulation of insulin resistance markers highlight the potential of hesperidin as a promising therapeutic intervention to counter the negative impact of liver insulin resistance caused by IL-6. These findings open avenues for further investigation into the mechanistic basis of hesperidin's anti-diabetic properties and its potential translational applications in managing insulin resistance in liver cells (Chae and Kim, 2012[14]).

Wang and coworkers conducted a study to explore the protective effects of naringenin and hesperetin on diabetic db/db mice. Hesperetin was administered at 50 mg/kg/day daily for six weeks to the diabetic db/db mice. The results showed that hesperetin, similar to naringenin, effectively protected pancreatic islets in diabetic mice. It led to the rescue of islet destruction, an increase in islet size and mass, improvement in glucose tolerance, elevation of insulin concentrations in the serum, and reduction in levels of fasting blood glucose in the db/db mice. These findings underscore the potential benefits of flavanones, dietary supplements containing compounds like naringenin and hesperetin, as well as phytomedicine abundant in flavanones for safeguarding β cells in the pancreas during late-stage diabetes (Wang et al., 2021[53]).

Additionally, Peng and coworkers demonstrated hesperidin's preventative influence on type 2 diabetes mellitus (T2DM) within a rat model displaying insulin resistance induced by alloxan and a high-fat diet (HFD). Rats received an oral dosage of 100 mg/kg of hesperidin for 35 days. Results showed that hesperidin improved fasting serum glucose without altering insulin levels, indicating improved insulin sensitivity and prevention of insulin resistance and diabetes. Oral glucose tolerance test results also demonstrated the hesperidin treatment for preventing impaired glucose tolerance. Hesperidin was found to regulate glycolysis and gluconeogenesis in the liver, enhancing glucokinase activity while reducing the activity of glucose-6-phosphatase and phosphoenolpyruvate carboxykinase. It also increased glucose uptake in rat adipocytes. These findings suggested that hesperidin activates the insulin receptor (IR)/phosphoinositide-dependent kinase 1 (PDK1) pathway, improving insulin sensitivity. The study concludes that hesperidin has a significant preventative impact on insulin resistance caused by a high-fat diet through the activation of the IR/PDK1 pathway. It may offer a natural approach to enhance metabolic well-being and mitigate diabetes-related risks, providing a potential diabetes prevention option without significant side effects (Peng et al., 2021[38]).

### Protective effects of hesperidin and hesperetin countering the complications of diabetes 

Diabetes mellitus (DM) is a chronic disorder that can be complex and may cause various complications, which include neuropathy, nephropathy, retinopathy, testicular alterations, psychiatric effects, cardiovascular complications, and Diabetic skin ulcers. In this context, DM is recognized as a silent destructor of health. Interestingly, protective properties of hesperidin and hesperetin countering some complications of DM have been noted (Table 3(Tab. 3); References in Table 3: Abd Elsamie et al., 2021[1]; Abdou and Abd Elkader, 2022[2]; Agrawal et al., 2014[3][4]; Aksu et al., 2021[9]; Ashafaq et al., 2014[10]; Bayir et al., 2023[12]; Chen et al., 2019[16]; El-Marasy et al., 2014[18]; Jagdish et al., 2010[27]; Kakadiya and Shah, 2010[29]; Kakadiya et al., 2010[28]; Kandemir et al., 2018[30]; Li et al., 2018[34]; Lim et al., 2022[35]; Liu et al., 2020[36]; Shi et al., 2012[48]; Wang et al., 2018[52]; Yassien and El-Ghazouly, 2021[56]; Yi et al., 2023[57]; Yin et al., 2017[58]; Zhang et al., 2018[60]; Zhu et al., 2020[62], 2023[61]). 

#### Diabetic-induced neuropathy 

Zhu and coworkers investigated the neuroprotective impacts of hesperidin on Neuro 2A (N2a) cells exposed to high glucose (HG). They found that hesperidin effectively protected N2a cells from HG-induced damage. N2a cells were exposed to HG conditions, and hesperidin was administered at different doses (5, 10, and 20 μM) for 48 hours. HG reduced cell viability and increased LDH release, indicating cell injury. However, hesperidin treatment at 10 and 20 μM restored cell viability and decreased LDH release. Moreover, hesperidin activated the nuclear factor erythroid 2-related factor 2 (Nrf2)/antioxidant-response element (ARE) signaling pathway, as evidenced by increased Nrf2 expression, reversed reductions in glyoxalase 1 (Glo-1) levels, and increased receptor for advanced glycation end products (RAGE) levels induced by HG. The neuroprotective effects of hesperidin were hindered when ML385 inhibited Nrf2. The findings suggest that hesperidin shields N2a cells from injury induced by HG through the activation of the Nrf2/ARE signaling pathway. Different doses of hesperidin (5, 10, and 20 μM) effectively provided neuroprotection against high glucose conditions in N2a cells (Zhu et al., 2020[62]).

Diabetic neuropathy poses a significant challenge in diabetes management. Lim et al. investigated how hesperidin protects neuronal cells from apoptosis due to high glucose levels. SH-SY5Y neuronal cells were exposed to high glucose levels-induced oxidative stress associated with neurodegenerative diseases. Hesperidin exhibited no toxicity at doses below 40 μM, and 20 μM was chosen as the optimal concentration. It effectively reduced reactive oxygen species (ROS) levels induced by high glucose and inhibited intracellular ROS increase dose-dependently. Hesperidin protected against glucose-induced DNA damage, suppressed endoplasmic reticulum (ER) stress, and exhibited anti-apoptotic effects. It reversed changes in pro- and anti-apoptotic protein levels, inhibited apoptotic effectors caspase-9 and caspase-3, and mitigated apoptotic body formation. In conclusion, hesperidin's potent antioxidant properties safeguarded neuronal cells from high glucose-induced oxidative injury, ER stress, and apoptosis. It also promoted cellular survival by inhibiting Jun N-terminal kinases (JNK) and extracellular signal−regulated kinase (ERK) signaling pathways induced by oxidative stress, suggesting its potential as a treatment for diabetic neuropathy (Lim et al., 2022[35]).

Bayir and coworkers explored the potential of hesperidin in alleviating diabetic neuropathy and elucidating the role of the transient receptor potential melastatin 2 (TRPM2) channels in this process. The hesperidin groups received a daily intragastric dose of 100 mg/kg for a period of 14 days. The findings indicated that hesperidin treatment in rats with diabetes effectively reduced STZ-induced thermal hyperalgesia and hyperglycemia. Additionally, histopathological analysis of the sciatic nerve revealed reduced damage with hesperidin treatment. Immunohistochemical analysis showed that treatment with hesperidin resulted in decreased immune activity of the TRPM2 channel, type 4 collagen and fibrinogen induced by STZ (Bayir et al., 2023[12]).

Additionally, the study investigated the activation of the TRPM2 channel in the mechanism underlying sciatic nerve injury of diabetic neuropathy rat model using the ELISA method. Based on the findings, it appears that hesperidin has a regulatory impact on increased reactive oxygen species (ROS), Poly (ADP-ribose) polymerase (PARP) 1, and activation of the TRPM2 channel in the sciatic nerves of rats modeled with diabetic neuropathy. Based on the findings, it seems that the treatment of hesperidin may alleviate neuropathy caused by diabetes through decreasing the activation of TRPM2 channels (Bayir et al., 2023[12]).

Another study investigated the therapeutic potential of hesperidin for use in the context of alleviating oxidative stress and its associated complications in the central nervous system (CNS) regarding the context of STZ-induced diabetes mellitus in rats. Rats developed diabetes mellitus through STZ induction, followed by oral administration of hesperidin (50 mg/kg body weight) once daily for four weeks. The results demonstrated that STZ-induced DM led to elevated markers of oxidative stress and reduced antioxidant activity in the brain. However, treatment with hesperidin effectively mitigated these alterations, highlighting its remarkable antioxidant and neuroprotective effects in the CNS. The findings indicate that hesperidin may hold promise as a potential therapeutic approach to combat oxidative stress-related CNS complications in diabetes (Ashafaq et al., 2014[10]).

#### Diabetic-induced nephropathy

Chen and coworkers evaluated the protective effects of hesperetin against diabetic nephropathy (DN). DN is a common complication that can occur with diabetes and involves the enzyme Glo-1 and alpha-carbonyl aldehydes. The researchers focused on understanding the underlying mechanisms of hesperetin's actions, particularly its impact on the Nrf2/ARE/Glo-1 pathway. The study involved administering doses of hesperetin (50 and 150 mg/kg) or tert-butylhydroquinone (TBHQ), an Nrf2 inducer, orally to diabetic rats for ten weeks. The results revealed significant improvements in renal function and structural changes in diabetic rats treated with hesperetin. Hesperetin upregulated Glo-1, effectively countering the advanced glycation end products (AGEs)/receptor for the advanced glycation end products (RAGE) axis and reducing inflammation. Also, hesperetin increased Nrf2 and p-Nrf2 levels and induced γ-glutamylcysteine synthetase, a gene regulated by the Nrf2/ARE pathway (Chen et al., 2019[16]).

Furthermore, hesperetin treatment reduced glomerular mesangial matrix expansion and decreased fibronectin (FN) and type IV collagen (CoIV) levels in the kidney. Ultrastructural improvements further supported its positive effects. Hesperetin also decreased the formation of AGEs and reduced pro-inflammatory cytokine levels. In conclusion, this study highlights the potential renoprotective impacts of hesperetin against DN in rats, achieved through modulation of the Nrf2/ ARE/Glo-1 pathway and suppression of AGEs and inflammation (Chen et al., 2019[16]).

Similarly, the treatment of hesperetin (at a dosage of 40 and 80 mg/kg for four weeks) effectively lowered levels of fasting blood glucose and enhanced the ability to tolerate glucose in mice that are diabetic due to STZ administration. Moreover, hesperetin exhibited significant kidney-protective effects by mitigating abnormalities in serum, liver, and kidney-related parameters. Notably, the compound reversed irregular distortions in the basement membrane of glomeruli and reduced the expansion of mesangial regions in the kidneys, thus preserving renal integrity. Furthermore, hesperetin plays a crucial role in the regulation of renal function. It increased the expression of renal nephrin, a protein essential for maintaining podocyte function while decreasing the renal alpha-smooth muscle actin (α-SMA) expression associated with kidney fibrosis. The protective effects of hesperetin were also attributed to its ability to suppress the presence of transforming growth factor-β1 (TGF-β1) and its subsequent influencers, Akt and integrin-linked kinase (ILK), in terms of their expression. This signaling pathway is recognized for its participation in advancing diabetic nephropathy (DN). Overall, the study highlights the potential therapeutic benefits of hesperetin in managing DN and safeguarding kidney health in diabetic individuals. Based on the results, it appears that hesperetin could be used as a promising natural compound for developing novel treatments to combat the debilitating effects of diabetic kidney disease (Zhang et al., 2018[60]).

Abdou et al. sought to explore the effects of hesperetin (40 mg/kg) in diabetic nephropathy (DN) induced by STZ using male albino rats. They demonstrated that the STZ-induced diabetic rats exhibited impaired kidney function, oxidative stress, and inflammation, as indicated by increased concentrations of urea, creatinine, BUN, inflammatory cytokines, and decreased levels of antioxidants. However, hesperetin treatment remarkably ameliorated these deleterious effects. Notably, hesperetin administration led to significant improvements in renal function, with a marked reduction in oxidative stress markers like Thiobarbituric acid reactive substances (TBARS), restoration of antioxidant levels such as total antioxidant capacity (TAC) and GSH, and enhanced enzymatic activity of CAT. Furthermore, hesperetin treatment effectively downregulated the expression of Glycogen synthase kinase-3β (GSK-3β), a key enzyme implicated in diabetic complications, offering protective effects against DN. The histopathological examination of renal tissue confirmed that hesperetin mitigated diabetes-induced damage, supporting its role in preserving kidney integrity. Overall, the comprehensive findings highlight the potential of hesperetin as a therapeutic agent to counter DN. Its multifaceted effects encompassing antioxidant, anti-inflammatory, and anti-diabetic properties make it a promising candidate for preventing and managing the complications associated with DN (Abdou and Abd Elkader, 2022[2]).

Additionally, Kandemir and coworkers explored hesperidin's hypoglycemic and antioxidant effects (200 mg/kg per day; orally over a period of four weeks) in the context of DN induced by STZ in rats. Diabetic rats exhibited increased urea, creatinine, and MDA levels and reduced antioxidant enzyme activities. Moreover, TGF-β1 level and 8-hydroxy-20-deoxyguanosine (8-OHdG) expression, indicative of DNA damage, were elevated, and histopathological changes in renal tissue were observed. Hesperidin treatment significantly attenuated these adverse effects, lowering serum urea and creatinine levels, decreasing MDA, and restoring antioxidant enzyme activities and GSH levels. Additionally, hesperidin reduced TGF-β1 and 8-OHdG levels, mitigating histopathological renal changes. Collectively, it may be suggested that hesperidin may hold promise as a potential treatment for DN (Kandemir et al., 2018[30]).

#### Diabetic-induced retinopathy

Shi and coworkers investigated the potential effects of hesperidin on retinal and plasma abnormalities in streptozotocin-induced diabetic rats. Diabetic retinopathy is a complex condition involving increased production of AGEs and elevated aldose reductase (AR) activity, leading to oxidative stress and inflammation. The researchers administered two doses of hesperidin (100 and 200 mg/kg) orally to diabetic rats for 12 weeks. The results demonstrated that hesperidin treatment attenuated blood-retina breakdown (BRB) and increased retinal thickness. Additionally, hesperidin significantly reduced blood glucose levels, AR activity, and concentrations of vascular endothelial growth factor (VEGF) in the retina, intercellular adhesion molecule-1 (ICAM-1), AGEs, IL-1β, and TNF-α. Moreover, hesperidin administration diminished levels of plasma MDA along with elevated activity of superoxide dismutase (SOD) in rats with diabetes. Based on these findings, it seems that hesperidin exerts beneficial effects through anti-angiogenic, anti-inflammatory, and antioxidative mechanisms. Furthermore, hesperidin's restraining impact on the accumulation of AGEs and the involvement of the polyol pathway in the retina may contribute to its potential in preventing diabetic retinopathy and improving metabolic health (Shi et al., 2012[48]).

#### Diabetic-induced testicular alterations

The addition of health could be advantageous in shielding diabetic rats from the decline in sperm quality and DNA damage in their testes induced by diabetes mellitus. In this regard, Aksu et al. evaluated various sperm parameters and oxidative stress markers in STZ-induced DM. In the DM group, sperm motility, mean cauda epididymis weights (MCEW), and DNA damage significantly decreased, while abnormal and dead sperm percentages significantly increased compared to the control group. Mean testis weights (MTW) showed no difference among the groups. The DM group also exhibited higher levels of MDA and 8-hydroxy-20-deoxyguanosine (8-OHdG), indicating increased oxidative stress. However, hesperidin treatment improved sperm motility and reduced DNA damage and MDA levels, though 8-OHdG levels remained higher than in the control and hesperidin groups. Intracellular antioxidant defense compounds (GSH, GPx, CAT, and SOD) were increased by hesperidin treatment, suggesting a potential protective effect against oxidative stress induced by DM (Aksu et al., 2021[9]).

Another study also assessed the potential of hesperidin in mitigating testicular alterations induced by diabetes in rats. The male reproductive organs are adversely affected by diabetes mellitus, leading to testicular atrophy and consequent infertility, mainly attributed to the harmful impact of hyperglycemia and oxidative stress. Throughout the experiment, diabetic rats were administered hesperidin orally at 200 mg/kg per day over a period of ten consecutive days after confirming hyperglycemia. The outcomes of this intervention were notably positive. Hesperidin effectively reduced blood glucose levels, reinstated normal body and testicular weights, and significantly ameliorated the histopathological changes in the testicular tissues (Abd Elsamie et al., 2021[1]).

Moreover, it played a vital role in restoring normal germinal epithelium and spermatogenesis, rectifying the morphological abnormalities observed in spermatozoa due to diabetes. Although insulin also exhibited some improvements in the adverse effects of diabetes, the results indicated that hesperidin was even more potent in counteracting the toxic impact of diabetes on the testes compared to insulin treatment. These promising findings suggest that hesperidin holds substantial therapeutic potential as a valuable agent in addressing diabetic complications in the male reproductive system, particularly testicular dysfunction (Abd Elsamie et al., 2021[1]).

#### Diabetic-induced psychiatric effects

El-Marasy and coworkers investigated the anti-depressant impacts of orally administered hesperidin on STZ-induced diabetic rats. Diabetic rats experienced significant body mass loss and elevated blood glucose levels compared to normal rats. Hesperidin administration at 25.0 mg/kg/day resulted in a significant gain in body mass, while 50 mg/kg/day and 100 mg/kg/day reduced body mass loss and caused a considerable loss in body mass, respectively. Similarly, fluoxetine (5 mg/kg/day) reduced body mass loss. Hesperidin administration at different doses markedly lowered blood glucose concentrations in rats with diabetes. Locomotor activity was reduced in all groups, with no significant difference compared to normal rats. Hesperidin and fluoxetine treatments significantly decreased immobility duration in diabetic rats. Diabetic rats had increased MDA brain levels, while hesperidin and fluoxetine administration showed varying effects on MDA levels. Brain levels of GSH, IL-6, brain-derived neurotrophic factor (BDNF), norepinephrine (NE), 5-hydroxytryptamine (5-HT), and dopamine (DA) were also altered in diabetic rats with different hesperidin and fluoxetine administration responses. Overall, the study highlights the potential of hesperidin in mitigating diabetic effects on body mass, blood glucose levels, immobility duration, oxidative stress, and neurotransmitter levels in rats (El-Marasy et al., 2014[18]). 

Another study also explored the neuroprotective effects of hesperidin (50 mg/kg and 150 mg/kg for ten weeks) in diabetic rats with depression-like behaviors. The researchers also used an Nrf2 inducer called tert-butylhydroquinone (TBHQ) (25 mg/kg) as a comparison. The findings revealed that hesperidin exhibited anti-depressant and anxiolytic effects in diabetic rats. It reduced immobility time in the forced swimming test and increased time spent in open areas during behavioral tests. Moreover, hesperidin enhanced the activity of Glo-1, a critical detoxifying enzyme. It inhibited the AGEs/RAGE axis and oxidative stress in the brain. Also, hesperidin increased the levels of Nrf2, a pivotal element in cellular protection against oxidative stress. This study suggests that hesperidin may hold promise as a potential therapeutic agent for mitigating depression-like behaviors in diabetic individuals. The observed neuroprotective effects of hesperidin are linked to its ability to regulate critical molecular pathways involved in oxidative stress and neuroprotection (Zhu et al., 2020[62]).

Similarly, Zhu et al. explored the anxiety-reducing impacts of hesperidin on behavior related to anxiety in rats with diabetes. They explored the fundamental mechanisms involving the pathway of protein kinase A (PKA)/cAMP response element-binding protein (CREB). Rats exhibiting diabetes induced by STZ were subjected to oral administration of hesperidin (50 and 150 mg/kg) over a period of ten weeks. Anxiety-like behaviors were assessed using various tests. The results showed that hesperidin supplementation produced anxiety-reducing effects in rats with diabetes, evidenced by improved behavior in elevated plus maze, hole board, and marble-burying tests. Hesperidin also increased the expression of CREB, BDNF, PKA, and the synaptic proteins within the hippocampus and amygdala of rats with diabetes, thus restoring the equilibrium within the pathway involving PKA/CREB/BDNF. *In vitro* experiments further verified that the protective effects of hesperidin were facilitated through the mediation of the PKA/CREB/ BDNF pathway. The study suggests that hesperidin may have therapeutic potential for treating anxiety-like behaviors associated with diabetes by modulating the PKA/CREB/ BDNF pathway (Zhu et al., 2023[61]).

#### Diabetic cardiovascular complications

Yi and coworkers investigated the impact of a combination of trans-resveratrol (tRES) and hesperetin on endothelial cells (ECs) derived from healthy and type 2 diabetic donors. After subjecting the ECs to tRES+ hesperetin treatment for 48 hours, a comprehensive proteomic analysis identified 179 proteins with significant differences between diabetic and healthy ECs, and 81 proteins displayed substantial changes following tRES+ hesperetin treatment in diabetic ECs. The altered proteins in diabetic ECs were associated with diverse cellular functions, including cytoplasmic and membrane localization, nucleus and cytoskeleton organization, and involvement in various biological processes and molecular activities. Among the identified proteins, 16 displayed reversed differences between diabetic and healthy ECs after tRES+ hesperetin treatment. These 16 proteins were found to be associated with essential cellular processes such as angiogenesis, blood vessel development, endothelial tube morphogenesis, and response to hypoxia (Yi et al., 2023[57]).

Further functional validation experiments revealed that tRES+ hesperetin treatment effectively targeted key proteins, including activin A receptor-like type 1 and transforming growth factor β receptor 2 (TGFBR2), critical in regulating angiogenesis. These results indicate that the tRES+ hesperetin combination profoundly influences the protein profiles of diabetic ECs, potentially mitigating the adverse effects of diabetes on endothelial function. Taken together, hesperetin may offer potential avenues for future therapeutic interventions to combat diabetic endothelial dysfunction (Yi et al., 2023[57]).

Kakadiya et al. evaluated the impact of hesperidin on cardiovascular complications in rats with diabetes provoked by STZ and nicotinamide administration. The rats received treatment with oral hesperidin at a dosage of 100 mg/kg administered daily for a duration of 28 days, subsequent to diabetes and myocardial infarction induction. The findings indicated that rats with diabetes induced by STZ-nicotinamide displayed elevated levels of serum HbA1c, creatine kinase (CK), glutamate oxaloacetate transferase (GOT), glycogen, and nitrite, along with a significant decrease in myocardial infarct size. However, the administration of hesperidin resulted in substantial reductions in HbA1c, glucose levels, nitrite, CK, and glycogen compared to the diabetic control group. Additionally, the histopathological analysis revealed that hesperidin treatment in diabetic rats led to reduced necrosis and fragmentation of muscle fibers, indicating a potential protective effect on the heart. In conclusion, the study suggests that hesperidin may alleviate cardiovascular complications in rats with type 2 diabetes. Hesperidin positively affected serum enzymes and myocardial tissue parameters, reducing myocardial infarct size and improving cardiac tissue appearance (Kakadiya and Shah, 2010[29]).

Similarly, another study aimed to assess the antioxidative impacts of hesperidin on myocardial infarction induced by isoproterenol in both non-diabetic and diabetic rats. After inducing diabetes using STZ and nicotinamide, rats were treated with hesperidin (100 mg/kg, p.o) for 28 days before causing myocardial infarction with isoproterenol. Diabetic rats treated with STZ-nicotinamide showed elevated serum HbA1c levels and elevated generation of MDA/lipid peroxidation (LPO) and nitrite content within the heart tissue. Moreover, critical oxidative stress biomarkers, including reduced GSH, CAT, and SOD, exhibited reduced activity compared to control rats. However, treatment with hesperidin showed significant improvements. It effectively restored GSH levels, enhanced CAT and SOD activity, and reduced nitrite levels and lipid peroxidation in comparison to diabetic reference groups. This study proposes that hesperidin may effectively alleviate oxidative stress in the heart during isoproterenol-induced myocardial infarction in type 2 diabetic rats (Kakadiya et al., 2010[28]).

Additionally, in characterizing type 2 diabetes, STZ-nicotinamide administration induced severe hyperglycemia and increased HbA1c levels in rats. Notably, hesperidin treatment, either alone or in combination, resulted in notable decreases in HbA1c levels and glucose compared to diabetic control subjects. Hesperidin demonstrated promising antioxidant properties, making it a potential therapeutic option for managing oxidative stress in the heart during diabetic myocardial infarction. Furthermore, the positive effects on glucose, HbA1c, and anti-oxidant levels suggest the promising effects of hesperidin against diabetes-induced myocardial infarction (Kakadiya et al., 2010[28]).

Liu and coworkers investigated the potential vasomotor effects of hesperetin (100 mg/kg/day for eight weeks) on the coronary arteries of rats impaired by diabetes or elevated glucose concentrations. The results demonstrated chronic hesperetin treatment relieved the heightened contractile sensitivity and reduced vasodilator responsiveness induced by diabetes in rat coronary arteries (RCAs). Additionally, hesperetin increased the expression of voltage-dependent K+ (Kv) 1.2 channels in diabetic RCAs, improving vasomotor function. Furthermore, the chronic administration of hesperetin showed several positive effects, including attenuating diabetes-induced body weight loss and reducing elevated plasma glucose levels. Furthermore, it reversed the hypersensitivity of diabetic RCAs and restored their responsiveness to relaxants. Moreover, hesperetin increased Kv currents in diabetic rat coronary arterial smooth muscle cells (RCASMCs) and upregulated the expression of Kv1.2 channels. To conclude, this research indicates the potential of hesperetin as a viable therapeutic option for managing vasomotor dysfunction in diabetic RCAs. Its beneficial effects on contractile and relaxant responses and upregulation of Kv1.2 channels indicate its potential for addressing vascular complications associated with diabetes in rat coronary arteries (Liu et al., 2020[36]).

Another study examined the potential cardioprotective impacts of hesperidin in the context of myocardial dysfunction in diabetic rats induced by isoproterenol. Rats with diabetes initiated by STZ were subjected to hesperidin treatment (100 mg/kg orally), the antagonist of PPAR-γ GW9662 (1 mg/kg intraperitoneal injection), or a mix of both compounds. The results of the study unveiled remarkable cardioprotective properties of hesperidin. Notably, hesperidin treatment enhanced hemodynamic functions, promoting optimal systolic, diastolic, and mean arterial pressures and supporting positive changes in the rate of pressure development and left ventricular end-diastolic pressure. Moreover, hesperidin fortified the endogenous antioxidant defense system, reducing oxidative stress and decreasing lipid peroxidation. These effects contributed to preserving the structural integrity of the myocardium and mitigating myocardial damage caused by isoproterenol in diabetic conditions (Agrawal et al., 2014[3]).

Additionally, hesperidin demonstrated significant anti-apoptotic effects, as indicated by heightened levels of Bcl-2 protein expression accompanied by diminished Bax protein expression. Overall, the findings from this study highlight the promising therapeutic value of hesperidin as a potential intervention for managing myocardial infarction in diabetic individuals. By modulating the PPAR-c pathway and exerting beneficial effects on heart function and antioxidant defense, hesperidin shows promise in mitigating the detrimental effects of isoproterenol on the heart in the context of diabetes (Agrawal et al., 2014[3]).

Agrawal et al. investigated the potential cardioprotective effects of hesperidin in diabetic rats undergoing cardiac ischemia and reperfusion (I/R) injury. The rats received hesperidin (at a daily dosage of 100 mg/kg), GW9662 (an antagonist targeting the PPAR-γ receptor), or a mix of both compounds for a duration of 14 days. Subsequently, the rats underwent coronary artery occlusion followed by reperfusion. Hesperidin pretreatment significantly improved cardiac function, reduced markers of cardiac injury such as CK-MB and LDH, and lowered lipid peroxidation levels. Moreover, hesperidin decreased the pro-inflammatory cytokine TNF-α, indicating its anti-inflammatory potential. The flavonoid also demonstrated anti-apoptotic properties through the elevation of Bcl-2 protein expression and reducing the expression of the pro-apoptotic protein Bax, highlighting its ability to inhibit cell death. Assessments of tissue histology and detailed structural analyses supported the safeguarding effect of hesperidin, as it mitigated myocardial damage and preserved mitochondrial structure. These results indicated that hesperidin has the potential to serve as a promising natural therapeutic option for protecting the heart against ischemic injury in diabetic individuals. Its effects were mediated through the activation of PPAR-γ receptors, as evidenced by the elevation of PPAR-γ protein levels. However, the presence of the PPAR-γ receptor antagonist, GW9662, dampened the beneficial effects of hesperidin, indicating the involvement of PPAR-γ signaling in mediating the cardioprotective properties of hesperidin (Agrawal et al., 2014[4]).

Another similar study determined the effects of hesperidin on complications related to the cardiovascular system within the context of myocardial infarction induced by isoproterenol in both non-diabetic and diabetic rats induced by nicotinamide and STZ administration. Hesperidin was administered orally at 100 mg/kg for 28 days. Diabetic rats exhibited elevated blood glucose, HbA1c, total cholesterol, LDL, triglycerides, and blood pressure. They reduced HDL, lecithin Cholesterol acyl transferase (LCAT), and lipoprotein lipase (LPL) levels. Myocardial infarction in diabetic rats further exacerbated these effects. However, treatment with hesperidin significantly reduced blood glucose, HbA1c, total cholesterol, LDL, triglycerides, and blood pressure, while increasing LCAT and LPL levels. These findings indicate that hesperidin may effectively control blood glucose concentrations and reduce cardiac complications in a laboratory-induced model of myocardial infarction among diabetic rats (Kakadiya et al., 2010[28]).

#### Diabetic skin ulcer

Wang and coworkers investigated the effects of hesperidin on STZ-induced diabetic foot ulcer wounds in rats. The rats with induced wounds received gradually increasing amounts of hesperidin orally (doses of 10 to 80 mg/kg were given) and insulin injected subcutaneously (at a dose of 10 IU/kg). According to the findings, it was revealed that hesperidin at 60 and 80 mg/kg doses significantly improved glucose, HbA1C, insulin concentration, and wound dimension. It also modulated the expression of VEGF and reduced the levels of TNF-α and IL-6, indicating reduced inflammation. Hesperidin administration led to increased wound healing in diabetic rats, and higher doses of hesperidin (80 mg/kg) showed better results in lowering serum glucose levels than in lower doses (Wang et al., 2018[52]).

Additionally, hesperidin administration improved serum insulin levels and reduced glycated Hb concentrations. It also increased the activity of antioxidant enzymes, such as SOD and GSH, and decreased MDA and MPO levels, indicating reduced oxidative stress. Hesperidin treatment also improved insulin resistance (Homeostasis model of insulin resistance (HOMA-IR) values) and reduced pro-inflammatory markers TNF-α and IL-6 levels. Furthermore, hesperidin administration upregulated the expression of VEGF and its receptors, VEGFR1 and VEGFR2, promoting angiogenesis and wound healing in diabetic foot ulcers. In conclusion, hesperidin showed promising effects on the process of healing in ulcers of diabetic foot by reducing inflammation, hyperglycemia, and oxidative stress and promoting angiogenesis. Higher doses of hesperidin (80 mg/kg) seemed to be more effective in improving the observed parameters in this study (Wang et al., 2018[52]).

A similar study evaluated the potential of hesperidin (25, 50, and 100 mg/kg for 21 days) in treating diabetes-induced foot ulcers in rats. Hesperidin treatment at 50 and 100 mg/kg significantly inhibited decreased body weight and increased blood glucose, food, and water intake induced by STZ. It also led to a notable rise in wound closure and insulin concentrations in the serum. Furthermore, hesperidin attenuated the altered levels of oxidative stress markers in the wound tissue, increasing SOD and GSH levels while reducing MDA and NO levels. Hesperidin treatment upregulated the mRNA expression of VEGF-c, angiopoietin-1 (Ang-1), Tie-2, TGF-β1, and suppressor of Mothers Against Decapentaplegic (SMAD) 2/3 in the wound tissue, suggesting enhanced angiogenesis and vasculogenesis. Histopathological examination revealed that hesperidin-treated rats exhibited improved wound architecture, increased blood vessels, re-epithelialization, and reduced leukocyte infiltration.

In conclusion, hesperidin demonstrated the potential to accelerate wound healing in chronic diabetic foot ulcers. It showed beneficial effects on body weight, glucose control, oxidative stress, and wound tissue remodeling. The upregulation of angiogenic and vasculogenic factors further supported hesperidin's wound-healing properties. Thus, hesperidin could be viewed as a potential and promising therapeutic choice for delayed wound healing associated with diabetes (Li et al., 2018[34]).

Another study evaluated the healing effect of hesperidin on diabetic skin injuries in male albino rats. The untreated diabetic rats showed incomplete wound closure, thickened and malformed epidermis, disorganized collagen fibers, and significant inflammation. Nonetheless, upon administering hesperidin treatment (orally administered at a dosage of 50 mg/kg for a period of 30 days) to diabetic rats, remarkable improvements were observed. The wounds closed entirely with a thin and normal-looking epidermis, well-organized collagen fibers, and enhanced angiogenesis, as indicated by strong positive VEGF immunoreactivity. These findings suggest that hesperidin can be a valuable adjunctive or alternative agent in promoting diabetic wound healing, leading to positive cosmetic outcomes. This research highlights the potential benefits of hesperidin derived from citrus herbal products in addressing skin ulcers and poor healing in diabetic patients (Yassien and El-Ghazouly, 2021[56]).

### Clinical investigations

Yari et al. carried out a randomized clinical trial with a placebo control group involving individuals at risk of developing diabetes. The study involved 48 participants, divided into two groups: one receiving a lifestyle intervention and a treatment combination of milled brown flaxseed (30 g) and hesperidin (two capsules, each containing 500 mg), along with lifestyle modifications, administered daily over a 12-week period. The aim was to assess changes in lipid profile, glucose homeostasis, inflammatory biomarkers, anthropometric measures, and atherogenic indices. Results indicated that the combined therapy group remarkably reduced body weight and blood pressure compared to the control group. The intervention group also showed improved lipid profiles, lower triglyceride and LDL cholesterol levels, and better atherogenicity indices. Glucose homeostasis parameters also exhibited positive trends, with enhanced insulin sensitivity.

Furthermore, the combined therapy led to a notable decrease in the inflammatory biomarker TNF-α. Participants adhered well to the intervention, and no adverse effects were reported. In conclusion, the co-administration of brown flaxseed and hesperidin, combined with lifestyle modification, displayed promising effects in managing metabolic abnormalities in prediabetic individuals, suggesting its potential as a practical approach for diabetes risk management (Yari et al., 2021[55]).

## Conclusion and Future Prospects

In summary, the collective evidence from the reviewed studies strongly supports the potential therapeutic utility of hesperidin and hesperetin in managing diabetes and its related complications. The observed molecular, cellular, and metabolic effects underscore the diverse mechanisms through which these compounds exert their beneficial impacts. Hesperetin has demonstrated its ability to modulate inflammatory cytokine release, NF-κB acetylation, and SIRT 3/6 expression through the TLR/MyD88/NF-κB signaling pathways, suggesting its promise as a treatment option for averting diabetes and its associated complications. On the other hand, hesperidin shows excellent potential as a biomolecule for treating diabetic neuropathy, with its neuroprotective effects achieved through enhancing Glo-1 and inhibiting the AGEs/ RAGE interaction via Nrf-2/ARE pathway activation.

Moreover, both hesperidin and hesperetin effectively normalize blood glucose levels by influencing glucose-regulating enzyme activity and reducing serum and liver lipid levels, making them viable candidates for hypoglycemic and hypolipidemic interventions in diabetes. Furthermore, the studies indicate that hesperidin may offer protective benefits in countering diabetic nephropathy through the inhibition of the signaling pathway involving TGF-β1, ILK, and Akt and improving renal function. Additionally, hesperidin exhibits antioxidant, anti-inflammatory, and anti-depressant effects in diabetic conditions, further expanding its potential therapeutic applications.

In conclusion, these findings strongly support considering hesperidin and hesperetin as potential complementary treatments for diabetes and its complications. However, further research and clinical studies are needed to fully understand the underlying mechanisms and validate their therapeutic efficacy in human subjects. These natural compounds hold promise for developing novel and cost-effective treatment options for individuals with diabetes.

## Notes

Amirhossein Mirzaei, Ali Mirzaei and Shakiba Najjar Khalilabad contributed equally as first author.

## Declaration

### Acknowledgment

This study was supported by Mashhad University of Medical Sciences, Mashhad, Iran. 

### Conflict of interest

The authors declare there is no conflict of interest. 

### Data availability

No data were used to support this study.

### Ethical statement

This is a review article. Ethical approval is not required for the study.

### Funding

None.

### Credit authorship contribution statement

Amirhossein Mirzaei: Investigation, resources, writing original draft; Ali Mirzaei: Investigation, resources, writing original draft; Shakiba Najjar Khalilabad: Investigation, resources, writing original draft; Vahid Reza Askari: Conceptualization, supervision, writing review & editing; Vafa Baradaran Rahimi: Conceptualization, project administration, supervision, writing review & editing.

## Figures and Tables

**Table 1:**
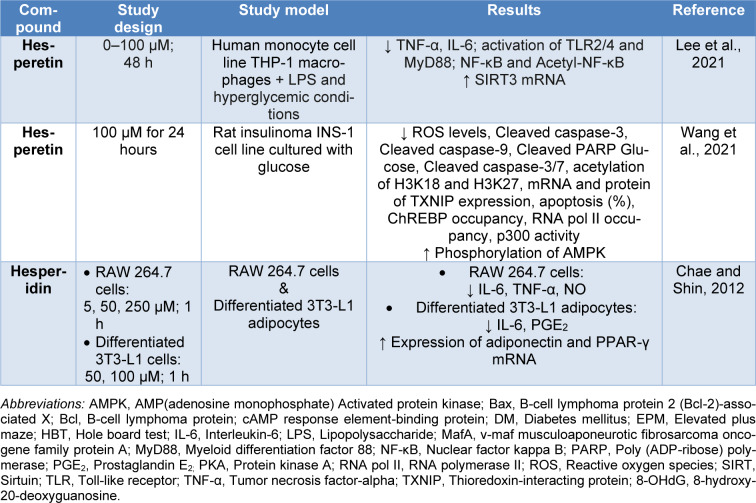
Protective effects of hesperidin and hesperetin against *in vitro* models of DM

**Table 2:**
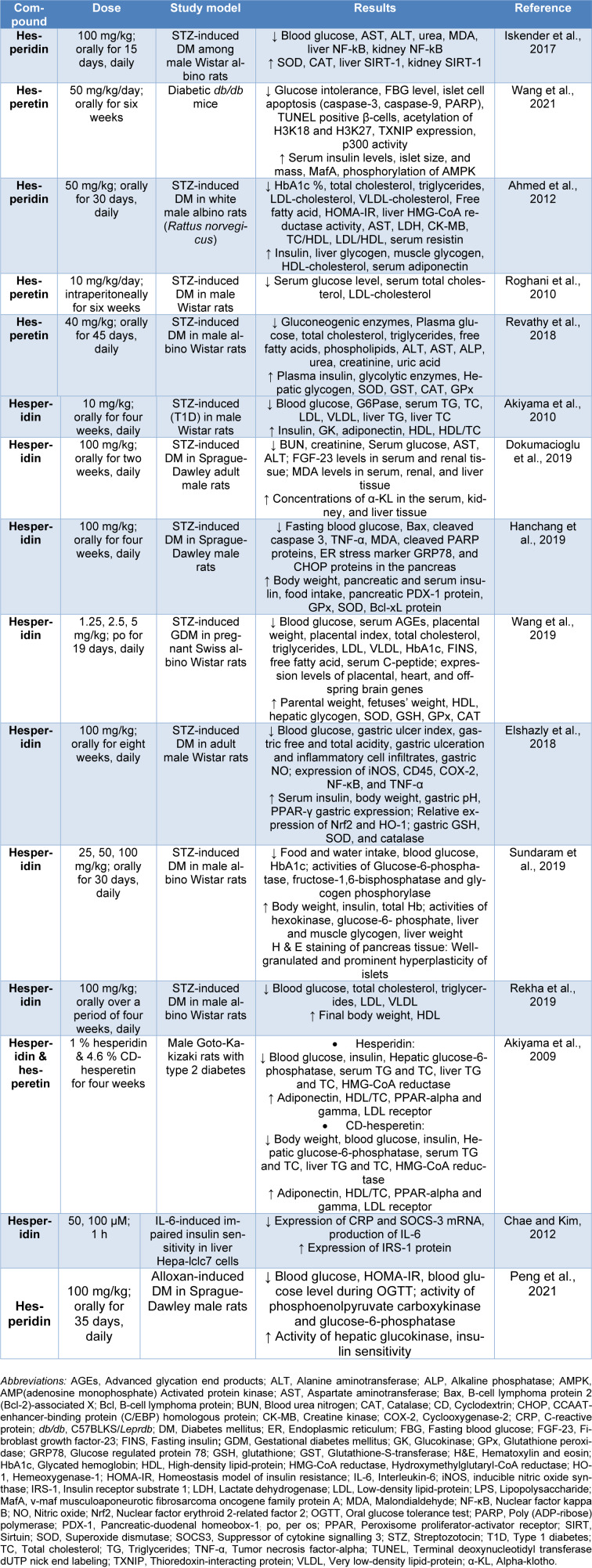
Protective effects of hesperidin and hesperetin against animal models of DM

**Table 3:**
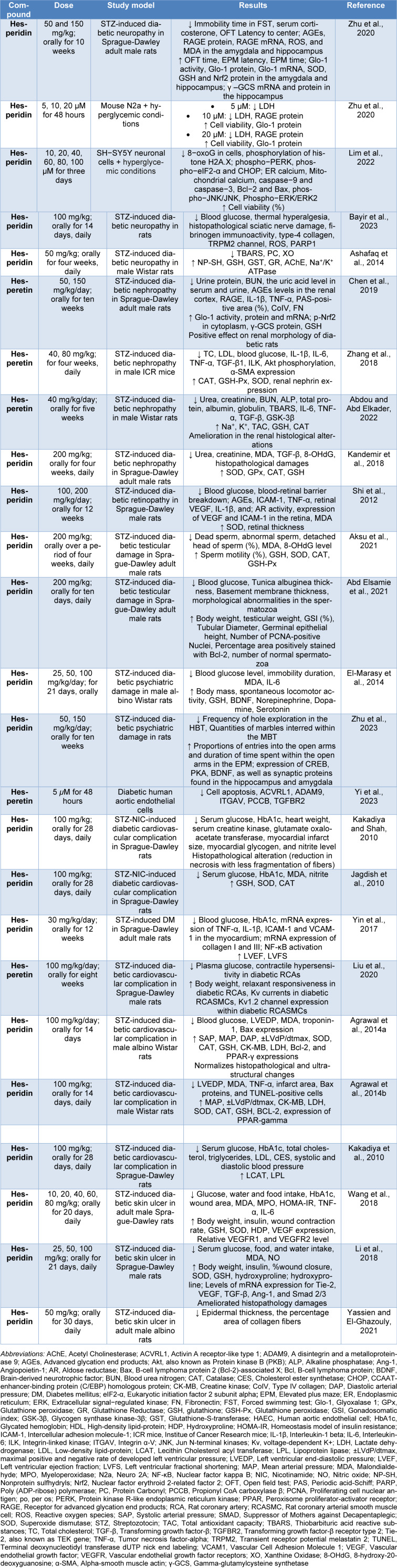
Protective effects of hesperidin and hesperetin against DM complications

## References

[1] Abd Elsamie MS, Ali MHM, Mahmoud OM, Kamel EM (2021). Influence of each hesperidin and insulin on diabetes-induced testicular alterations in adult albino rats. Eur J Anat.

[2] Abdou HM, Abd Elkader HTA (2022). The potential therapeutic effects of Trifolium alexandrinum extract, hesperetin and quercetin against diabetic nephropathy via attenuation of oxidative stress, inflammation, GSK-3 beta and apoptosis in male rats. Chem Biol Interact.

[3] Agrawal YO, Sharma PK, Shrivastava B, Arya DS, Goyal SN (2014). Hesperidin blunts streptozotocin-isoproternol induced myocardial toxicity in rats by altering of PPAR-gamma receptor. Chem Biol Interact.

[4] Agrawal YO, Sharma PK, Shrivastava B, Ojha S, Upadhya HM, Arya DS (2014). Hesperidin produces cardioprotective activity via ppar-gamma pathway in ischemic heart disease model in diabetic rats. PloS One.

[5] Ahmed OM, Mahmoud AM, Abdel-Moneim A, Ashour MB (2012). Antidiabetic effects of hesperidin and naringin in type 2 diabetic rats. Diabetologia Croatica.

[6] Akhlaghipour I, Nasimi Shad A, Askari VR, Maharati A, Baradaran Rahimi V (2023). How caffeic acid and its derivatives combat diabetes and its complications: A systematic review. J Funct Foods.

[7] Akiyama S, Katsumata S, Suzuki K, Ishimi Y, Wu J, Uehara M (2010). Dietary hesperidin exerts hypoglycemic and hypolipidemic effects in streptozotocin-induced marginal type 1 diabetic rats. J Clin Biochem Nutr.

[8] Akiyama S, Katsumata SI, Suzuki K, Nakaya Y, Ishimi Y, Uehara M (2009). Hypoglycemic and hypolipidemic effects of hesperidin and cyclodextrin-clathrated hesperetin in Goto-Kakizaki rats with type 2 diabetes. Bioscience, Biotechnol Biochem.

[9] Aksu EH, Kandemir FM, Kucukler S (2021). Ameliorative effect of hesperidin on streptozotocin-diabetes mellitus-induced testicular DNA damage and sperm quality degradation in Sprague-Dawley rats. J Food Biochem.

[10] Ashafaq M, Varshney L, Khan MH, Salman M, Naseem M, Wajid S (2014). Neuromodulatory effects of hesperidin in mitigating oxidative stress in streptozotocin induced diabetes. Biomed Res Int.

[11] Askari VR, Baradaran Rahimi V, Assaran A, Iranshahi M, Boskabady MH (2020). Evaluation of the anti-oxidant and anti-inflammatory effects of the methanolic extract of Ferula szowitsiana root on PHA-induced inflammation in human lymphocytes. Drug Chem Toxicol.

[12] Bayir MH, Yıldızhan K, Altındağ F (2023). Effect of hesperidin on sciatic nerve damage in STZ-induced diabetic neuropathy: modulation of TRPM2 channel. Neurotox Res.

[13] Buzdağlı Y, Eyipınar CD, Kacı FN, Tekin A (2022). Effects of hesperidin on anti-inflammatory and antioxidant response in healthy people: a meta-analysis and meta-regression. Int J Environ Health Res.

[14] Chae BS, Kim DK (2012). Hesperidin improves the IL-6-mediated hepatic insulin resistance in Hepa-1c1c7 cells. Nat Prod Sci.

[15] Chae BS, Shin TY (2012). Hesperidin ameliorates TNF-α-mediated insulin resistance in differentiated 3T3-L1 cells. Nat Prod Sci.

[16] Chen YJ, Kong L, Tung ZZ, Zhang YM, Liu Y, Wang TY (2019). Hesperetin ameliorates diabetic nephropathy in rats by activating Nrf2/ ARE/glyoxalase 1 pathway. Biomed Pharmacother.

[17] Dokumacioglu E, Iskender H, Musmul A (2019). Effect of hesperidin treatment on alpha-Klotho/FGF-23 pathway in rats with experimentally-induced diabetes. Biomed Pharmacother.

[18] El-Marasy SA, Abdallah HM, El-Shenawy SM, El-Khatib AS, El-Shabrawy OA, Kenawy SA (2014). Anti-depressant effect of hesperidin in diabetic rats. Canadian journal of physiology and pharmacology.

[19] Elshazly SM, El Motteleb DM, Ibrahim I (2018). Hesperidin protects against stress induced gastric ulcer through regulation of peroxisome proliferator activator receptor gamma in diabetic rats. Chem Biol Interact.

[20] Evans JA, Mendonca P, Soliman KFA (2022). Neuroprotective effects and therapeutic potential of the citrus flavonoid hesperetin in neurodegenerative diseases. Nutrients.

[21] Franke SI, Molz P, Mai C, Ellwanger JH, Zenkner FF, Horta JA (2018). Influence of hesperidin and vitamin C on glycemic parameters, lipid profile, and DNA damage in rats treated with sucrose overload. An Acad Bras Cienc.

[22] Garg A, Garg S, Zaneveld LJ, Singla AK (2001). Chemistry and pharmacology of the Citrus bioflavonoid hesperidin. Phytother Res.

[23] Ghadiri M, Baradaran Rahimi V, Moradi E, Hasanpour M, Clark CCT, Iranshahi M (2021). Standardised pomegranate peel extract lavage prevents postoperative peritoneal adhesion by regulating TGF-β and VEGF levels. Inflammopharmacology.

[24] Gholoobi A, Askari VR, Naghedinia H, Ahmadi M, Vakili V, Baradaran Rahimi V (2021). Colchicine effectively attenuates inflammatory biomarker high-sensitivity C-reactive protein (hs-CRP) in patients with non-ST-segment elevation myocardial infarction: a randomised, double-blind, placebo-controlled clinical trial. Inflammopharmacology.

[25] Hanchang W, Khamchan A, Wongmanee N, Seedadee C (2019). Hesperidin ameliorates pancreatic beta-cell dysfunction and apoptosis in streptozotocin-induced diabetic rat model. Life Sci.

[26] Iskender H, Dokumacioglu E, Sen TM, Ince I, Kanbay Y, Saral S (2017). The effect of hesperidin and quercetin on oxidative stress, NF-kappa B and SIRT1 levels in a STZ-induced experimental diabetes model. Biomed Pharmacother.

[27] Jagdish K, Mehul S, Nehal S (2010). Effect of hesperidin on serum glucose, hba1c and oxidative stress in myocardial tissue in experimentally induced myocardial infarctionin diabetic rats. Pharmacognosy J.

[28] Kakadiya J, Mulani H, Shah N (2010). Protective effect of hesperidin on cardiovascular complication in experimentally induced myocardial infarction in diabetes in rats. J Basic Clin Pharm.

[29] Kakadiya J, Shah N (2010). Effect of hesperidin on cardiovascular complication in streptozotocin- nicotinamide induced type 2 diabetic rats. Int J Pharm Pharm Sci.

[30] Kandemir FM, Ozkaraca M, Kucukler S, Caglayan C, Hanedan B (2018). Preventive effects of hesperidin on diabetic nephropathy induced by streptozotocin via modulating TGF-1 and oxidative DNA damage. Toxin Rev.

[31] Khorasanian AS, Fateh ST, Gholami F, Rasaei N, Gerami H, Khayyatzadeh SS (2023). The effects of hesperidin supplementation on cardiovascular risk factors in adults: a systematic review and dose-response meta-analysis. Front Nutr.

[32] Kumar M, Dahiya V, Kasala ER, Bodduluru LN, Lahkar M (2017). The renoprotective activity of hesperetin in cisplatin induced nephrotoxicity in rats: Molecular and biochemical evidence. Biomed Pharmacother.

[33] Lee A, Gu H, Gwon MH, Yun JM (2021). Hesperetin suppresses LPS/high glucose-induced inflammatory responses via TLR/MyD88/NF-kappa B signaling pathways in THP-1 cells. Nutr Res Pract.

[34] Li W, Kandhare AD, Mukherjee AA, Bodhankar SL (2018). Hesperidin, a plant flavonoid accelerated the cutaneous wound healing in streptozotocin-induced diabetic rats: Role of TGF-ß/Smads and Ang-1/Tie-2 signaling pathways. EXCLI J.

[35] Lim C, Zhen AX, Ok S, Fernando PDSM, Herath HMUL, Piao MJ (2022). Hesperidin protects SH−SY5Y neuronal cells against high glucose−induced apoptosis via regulation of MAPK signaling. Antioxidants.

[36] Liu Y, Zhang L, Dong LN, Song QY, Guo PM, Wang Y (2020). Hesperetin improves diabetic coronary arterial vasomotor responsiveness by upregulating myocyte voltage-gated K(+)channels. Exp Ther Med.

[37] Majety P, Lozada Orquera FA, Edem D, Hamdy O (2023). Pharmacological approaches to the prevention of type 2 diabetes mellitus. Front Endocrinol (Lausanne).

[38] Peng P, Jin J, Zou GL, Sui YB, Han YB, Zhao DP (2021). Hesperidin prevents hyperglycemia in diabetic rats by activating the insulin receptor pathway. Exp Ther Med.

[39] Pyrzynska KJN (2022). Hesperidin: A review on extraction methods, stability and biological activities. Nutrients.

[40] Rahmani AH, Babiker AY, Anwar S (2023). Hesperidin, a bioflavonoid in cancer therapy: a review for a mechanism of action through the modulation of cell signaling pathways. Molecules.

[41] Rakhshandeh H, Rajabi Khasevan H, Saviano A, Mahdinezhad MR, Baradaran Rahimi V, Ehtiati S (2022). Protective effect of portulaca oleracea on streptozotocin-induced type i diabetes-associated reproductive system dysfunction and inflammation. Molecules.

[42] Rekha SS, Pradeepkiran JA, Bhaskar M (2019). Bioflavonoid hesperidin possesses the anti-hyperglycemic and hypolipidemic property in STZ induced diabetic myocardial infarction (DMI) in male Wister rats. J Nutr Intermediary Metab.

[43] Revathy J, Srinivasan S, Abdullah SHS, Muruganathan U (2018). Antihyperglycemic effect of hesperetin, a citrus flavonoid, extenuates hyperglycemia and exploring the potential role in antioxidant and antihyperlipidemic in streptozotocin-induced diabetic rats. Biomed Pharmacother.

[44] Roghani M, Baluchnejadmojarad T, Roghani-Dehkordi F (2010). Antihyperglycemic and antihyperlipidemic effect of chronic administration of hesperetin in diabetic rats. J Babol Univ Med Sci.

[45] Roohbakhsh Y, Baradaran Rahimi V, Silakhori S, Rajabi H, Rahmanian-Devin P, Samzadeh-Kermani A (2020). Evaluation of the effects of peritoneal lavage with rosmarinus officinalis extract against the prevention of postsurgical-induced peritoneal adhesion. Planta Med.

[46] Russo MP, Grande-Ratti MF, Burgos MA, Molaro AA, Bonella MB (2023). Prevalence of diabetes, epidemiological characteristics and vascular complications. Arch Cardiol Mex.

[47] Shams-Rad S, Mohammadi M, Ramezani-Jolfaie N, Zarei S, Mohsenpour M, Salehi-Abargouei A (2020). Hesperidin supplementation has no effect on blood glucose control: A systematic review and meta-analysis of randomized controlled clinical trials. Br J Clin Pharmacol.

[48] Shi XP, Liao S, Mi HJ, Guo CR, Qi DL, Li F (2012). Hesperidin prevents retinal and plasma abnormalities in streptozotocin-induced diabetic rats. Molecules (Basel, Switzerland).

[49] Sundaram R, Nandhakumar E, Banu HH (2019). Hesperidin, a citrus flavonoid ameliorates hyperglycemia by regulating key enzymes of carbohydrate metabolism in streptozotocin-induced diabetic rats. Toxicol Mech Meth.

[50] Sweeting A, Wong J, Murphy HR, Ross GP (2022). A clinical update on gestational diabetes mellitus. Endocr Rev.

[51] Vanderniet JA, Jenkins AJ, Donaghue KC (2022). Epidemiology of type 1 diabetes. Curr Cardiol Rep.

[52] Wang L, He T, Fu AD, Mao ZJ, Yi L, Tang S (2018). Hesperidin enhances angiogenesis via modulating expression of growth and inflammatory factor in diabetic foot ulcer in rats. Eur J Inflammation.

[53] Wang SW, Sheng H, Bai YF, Weng YY, Fan XY, Zheng F (2021). Inhibition of histone acetyltransferase by naringenin and hesperetin suppresses Txnip expression and protects pancreatic beta cells in diabetic mice. Phytomedicine.

[54] Wang Y, Wang LN, Xu GM, Wei DH (2019). Hesperidin exerts the gestational diabetes mellitus via AGEs-RAGE signalling pathway. Int J Pharmacol.

[55] Yari Z, Naser-Nakhaee Z, Karimi‐Shahrbabak E, Cheraghpour M, Hedayati M, Mohaghegh SM (2021). Combination therapy of flaxseed and hesperidin enhances the effectiveness of lifestyle modification in cardiovascular risk control in prediabetes: a randomized controlled trial. Diabetol Metab Syndr.

[56] Yassien RI, El-Ghazouly DES (2021). The role of hesperidin on healing an incised wound in an experimentally induced diabetic adult male albino rats. Histological and immunohistochemical study. Egypt J Histol.

[57] Yi Z, Wang JM, Mestareehi A, Li H, Zhang X, Venkata SPM (2023). Quantitative proteomics reveals transforming growth factor β receptor targeted by resveratrol and hesperetin coformulation in endothelial cells. ACS Omega.

[58] Yin YW, Xu YC, Ma HX, Tian XH (2017). Hesperetin ameliorates cardiac inflammation and cardiac fibrosis in streptozotocin-induced diabetic rats by inhibiting NF-kappa B signaling pathway. Biomed Res India.

[59] Yoshida Y, Wang J, Zu Y, Fonseca VA, Mauvais-Jarvis F (2023). Rising prediabetes, undiagnosed diabetes, and risk factors in young women. Am J Prev Med.

[60] Zhang YH, Wang B, Guo F, Li ZZ, Qin GJ (2018). Involvement of the TGF beta 1-ILK-Akt signaling pathway in the effects of hesperidin in type 2 diabetic nephropathy. Biomed Pharmacother.

[61] Zhu X, Liu H, Deng Z, Yan C, Liu Y, Yin X (2023). Hesperidin exerts anxiolytic-like effects in rats with streptozotocin- induced diabetes via PKA/CREB signaling. Curr Mol Pharmacol.

[62] Zhu X, Liu HY, Liu Y, Chen YJ, Liu YW, Yin XX (2020). The antidepressant-like effects of hesperidin in streptozotocin-induced diabetic rats by activating Nrf2/ ARE/glyoxalase 1 pathway. Front Pharmacol.

